# Clinical outcomes of Gaelic Athletic Association athletes after surgical stabilization in the setting of anterior shoulder instability

**DOI:** 10.1016/j.jseint.2021.11.006

**Published:** 2021-12-17

**Authors:** Martin S. Davey, Eoghan T. Hurley, Hannan Mullett

**Affiliations:** Sports Surgery Clinic, Dublin, Ireland

**Keywords:** GAA, Gaelic Athletic Association, RTP, Return to play, Bankart repair, Latarjet, Shoulder stabilization

## Abstract

**Background:**

Gaelic Athletic Association (GAA) games are collision sports played at an amateur level, which represent the most popular sports played on the island of Ireland. Each year, many GAA players in Ireland require surgical stabilization with either arthroscopic Bankart repair (ABR) or open Latarjet (OL) procedures in the setting of anterior shoulder instability. The purpose of this study was to evaluate the clinical outcomes, recurrence, and return to play (RTP) in athletes who play GAA games having undergone surgical stabilization with either ABR or OL procedures in the setting of anterior shoulder instability.

**Methods:**

A retrospective review of all patients with anterior shoulder instability whom had stabilization with either ABR or OL under a single surgeon between 2012 and 2018 was performed. Patients who were athletes partaking in GAA sports were followed up by chart review and telephone survey to assess their clinical outcomes including satisfaction, pain as measured on the visual analog scale score, the Subjective Shoulder Value, recurrence, complications, and revision surgeries. In addition, RTP rates, time to RTP, level of RTP, and Shoulder Instability–Return to Sport after Injury scores were evaluated.

**Results:**

A total of 200 GAA athletes (194 males) with a mean age of 23.9 ± 6.1 years with mean follow-up of 50.4 ± 24 months were included in this study. A total of 98.1% patients were satisfied with their procedure at the latest follow-up, with an overall recurrence rate of 5%. A total of 6.5% of athletes required revision surgery, of whom 4% required revision stabilization (all of whom had recurrence). The overall rate of RTP was 88% at mean 6.0 ± 1.7 months postoperatively, with 75% of athletes returning at the same or higher levels than their preinjury level. There were no significant differences for all outcome measures analyzed between patients who had ABR or OL procedures.

**Conclusion:**

GAA athletes with anterior shoulder instability treated with either ABR or OL procedures report excellent clinical outcomes at medium-term follow-up, with high satisfaction rates, excellent functional outcomes, and high rates of RTP. Furthermore, this cohort demonstrates low rates of recurrence after stabilization with few requiring revision surgery.

The shoulder is the most commonly dislocated joint in the body, with anterior shoulder instability affecting up to 2% of the general population.^16^^,^^27^ However, the incidence of anterior shoulder instability has been found to affect up to 15% of collision athletes, with many attending specialist shoulder surgeons for consultation on optimal management.^15^ As both arthroscopic Bankart repair (ABR) and open Latarjet (OL) procedures have been shown to result in excellent clinical outcomes in the long term, collision athletes often elect for surgical stabilization in the setting of anterior shoulder instability.^11^, ^12^, ^13^^,^^20^

Although much literature in relation to anterior shoulder instability in collision athletes focuses on athletes partaking in American football, hockey, and rugby, Gaelic Athletic Association (GAA) games such as Gaelic football and hurling are collision sports claimed to be the most commonly played sports on the island of Ireland.^2^^,^^10^^,^^22^^,^^25^ However, GAA games have increasing popularity with over 400 clubs established worldwide outside of Ireland, leading to over 50,000 registered GAA players in the United States alone.^26^ In their previous study, Clesham and Shannon reported excellent functional outcomes with a moderate recurrence rate after ABR in 31 GAA athletes at medium-term follow-up.^5^ To the best knowledge of the authors of this study, the aforementioned study is the only study in the literature reporting outcomes of GAA athletes after surgical stabilization, with no series at present reporting outcomes of GAA athletes after OL procedure.

The purpose of this study was to evaluate the clinical outcomes, recurrence, and return to play (RTP) in athletes who play GAA games having undergone surgical stabilization with either ABR or OL procedures in the setting of anterior shoulder instability. Our hypothesis was that GAA athletes who present with anterior shoulder instability would report excellent functional outcomes, high rates of RTP, and low recurrence rates after surgical stabilization with either ABR or OL.

## Methods

### Data collection

Having been granted ethical approval from our institutional review board, a retrospective review of medical notes was carried out on all patients who underwent either ABR or OL procedures over an 8-year period between 2012 and 2018 by a single board-certified fellowship-trained shoulder surgeon. The inclusion criteria for this study included (1) traumatic anterior labral lesion requiring stabilization surgery with either ABR or OL, (2) minimum 24-month follow-up, and (3) GAA athlete. The exclusion criteria for this study included (1) prior surgery on the ipsilateral shoulder, (2) extension of the labral lesion to posterior, superior labrum anterior-posterior lesions requiring repair, or (3) the requirement for biceps tenotomy or tenodesis during the index procedure. Clinical outcomes of interest included (1) the visual analog scale score for residual pain, (2) the Subjective Shoulder Value score,^9^ (3) recurrence of dislocations, (4) revision surgeries, and (5) complications. Furthermore, outcomes of interest relating to RTP for the GAA players included (1) level of RTP, (2) timing of RTP, (3) the Shoulder Instability–Return to Sport after Injury (SIRSI) score,^8^ and (4) in cases of no RTP or change of sport, whether ipsilateral shoulder issues or other factors were causative was noted.

### Surgical technique

Preoperative decision-making in relation to choice of surgical intervention was made on a patient-by-patient basis, with numerous factors being considered, including (1) magnetic resonance arthrography findings including (a) percentage of glenoid bone loss, as well as (b) concomitant injury to both the humeral head and the glenoid labrum; (2) patient instability status (high- vs. low-performance athletes, or those whom suffer from primary or recurrent instability), and (3) patient preference.^1^^,^^6^

All surgeries were performed in beach chair position under general anesthesia for both procedures. An examination under anesthesia was performed on both shoulders to evaluate instability, range of motion, and joint laxity. Arthroscopic examination was performed through a standard posterior portal including evaluation of the capsuloligamentous complex, while the glenoid and humerus were checked for osteochondral or osseous defects. A dynamic examination was performed to evaluate instability, laxity, and engagement of any osseous defects while moving the shoulder through its full range of motion. A probe was then used to assess the stability of the labrum and biceps anchor.

In the case of an ABR, the labrum was then mobilized, and the glenoid bone freshened. The capsulolabral tissues are fixed to the glenoid rim with suture anchors approximately up to the 11- or 1-o’clock position, respectively. The capsulolabral tissues were repaired with at least two 2.3-mm suture anchors (Osteoraptor; Smith & Nephew, London, U.K.). All arthroscopic knots were positioned away from the joint to avoid glenohumeral irritation.

In the case of an OL, after arthroscopic examination, a 4-cm-long skin incision is placed in extension of the axillary fold, starting approximately 2-3 fingerbreadths distal to the tip of the coracoid. A horizontal subscapularis-split was performed at the junction between its middle- and lower-third to expose the capsule. The coracoid graft was fixed to the glenoid with 2 standard 3.5-mm, partially threaded, cancellous screws. The graft was then contoured to be flush with the glenoid surface using a high-speed burr. Capsular closure was then performed with two to three 2-0 Ethibond sutures (J&J, Belgium), with 2-3 nonabsorbable sutures used to repair the subscapularis split. A plain film radiograph of the operated shoulder is requested as routine in the recovery suite immediately postoperatively.

### Rehabilitation and return to play

Postoperatively, the shoulder was placed in a sling for 3 weeks, while allowing nonresisted activities of daily living without elevation of the shoulder. Patients immediately began physiotherapy, which continuously increased in intensity over the next 9 weeks. Return to contact drills at GAA training was permitted after 12 weeks, whereas return to full-contact GAA practice and competitive games at club and intercounty level usually would follow within the next 3 months, depending on ongoing review of progress by either our institutional or the GAA team physiotherapist.

### Statistical analysis

Quantitative statistical analysis was performed using SPSS version 22 (IBM, Armonk, NY, USA). In cases of categorical or dichotomous variable analysis, Fisher’s exact test in the form of a 2 × 2 contingency table was used to evaluate for significant differences between groups. In contrast to continuous variable analysis, unpaired t-tests were used to evaluate for significant differences between groups. Only *P* values that equated to .05 or less were considered to be statistically significant.

## Results

### Patient demographics

A total of 200 athletes (194 males) with a mean age of 23.9 ± 6.1 years and mean follow-up duration of 50 ± 24 months were included in this study. Of these, 148 (74%), 39 (19.5%), and 13 (6.5%) played Gaelic football, hurling, or both sports, respectively. A total of 103 and 97 GAA athletes underwent ABR and OL procedures, respectively. A summary of patient demographics is further illustrated in Table I.

**Table I:** Patient demographics.

Procedure	ABR	OL	*P* value
N GAA players	103	97	N/a
N Gaelic football (%)	80 (77.7)	68 (70.1)	.260
N Hurling (%)	15 (14.6)	24 (24.7)	.077
N Dual players† (%)	8 (7.7)	5 (5.2)	.570
N Males (%)	98 (95.1)	96 (98.9)	.213
Mean age ± SD (yr)	24.7 ± 7.3	23.1 ± 4.8	.070
Mean F/U ± SD (mo)	62 ± 26	38 ± 21	.0001∗

*ABR*, arthroscopic Bankart repair; *F/U*, follow-up; *GAA*, Gaelic Athletic Association; *N/a*, not applicable; *OL*, open Latarjet; *SD*, standard deviation.

∗ *P* < .0001.

† Dual player participating in Gaelic football and hurling.

### Clinical outcomes

The mean Subjective Shoulder Value scores reported for the GAA athletes who underwent ABR and OL procedures were 86.5 ± 19.2 and 85.9 ± 14.4 (*P* = .804), with mean visual analog scale scores of 1.7 ± 1.9 vs. 2.1 ± 2.0, respectively (*P* = .149). The overall patient-reported satisfaction rates for GAA athletes undergoing ABR and OL procedures were 98.1% and 98.0%, respectively (*P* > .99).

The overall recurrence rate for GAA athletes after surgical stabilization for anterior shoulder instability was 5.0% (10/200); 80% of whose injuries occurred while playing GAA at a mean of 18.7 months after stabilization. The total rate of recurrence after ABR was 3.8% at a mean follow-up duration of 17.7 ± 8.9 months (10-30) vs. the total rate of recurrence after OL of 6.2% at a mean follow-up duration of 19.7 ± 9.6 months (10-36).

The overall rate of surgical revisions for GAA athletes after surgical stabilization for anterior shoulder instability was 6.5% (13/200), of which 61.5% required revision stabilization. A total of 5 GAA athletes (4.9%) required surgical revisions after ABR procedures, of which 2 required revision in an OL procedure for recurrence, 2 underwent arthroscopic rotator cuff repairs, and 1 underwent an arthroscopic subacromial decompression. In contrast, a total of 8 GAA athletes (8.2%) required surgical revisions after OL procedures, of which 6 required a further open stabilization procedure for recurrence, 1 underwent reverse shoulder arthroplasty, and 1 underwent removal of metal.

A summary of these clinical findings is further illustrated in Table II.

**Table II:** Clinical outcomes.

Procedure	ABR	OL	*P* value
N Total	103	97	N/a
Satisfaction	101 (98.1%)	95 (98.0%)	>.99
Would undergo surgery again	98 (95.1%)	93 (95.9%)	>.99
SSV ± SD	86.5 ± 19.2	85.9 ± 14.4	.804
VAS ± SD	1.7 ± 1.9	2.1 ± 2.0	.149
Recurrence	4 (3.8%)	6 (6.2%)	.528
Recurrence playing GAA	4 (100%)	4 (67.7%)	>.99
Mean time (mo) to recurrence (range)	17.7 (10-30)	19.7 (10-36)	.641
Revision surgeries	5 (4.9%)	8 (8.2%)	.397
Further stabilization	2 (1.9%)	6 (6.2%)	.160
Other procedure	3 (3.0%)	2 (2.0%)	.213

*ABR*, arthroscopic Bankart repair; *GAA*, Gaelic Athletic Association; *OL*, open Latarjet; *SD*, standard deviation; *SSV*, Subjective Shoulder Value; *VAS*, Visual Analogue Scale.

### Return to play

The overall rate of RTP for GAA athletes after surgical stabilization for anterior shoulder instability was 88.0% (176/200), with 75% (150/200) returning to their preinjury level or higher. For those who underwent ABR (n = 103), the rate of RTP was 86.4% at a mean 5.7 months after ABR, with 71.8% returning at their preinjury level or higher. In contrast for those who underwent OL (n = 97), the rate of RTP was 89.7% at a mean 6.2 months after ABR, with 78.4% returning at their preinjury level or higher. The mean SIRSI scores for those GAA athletes who underwent ABR and OL procedures were 86.7 ± 19.4 and 85.9 ± 24.1, respectively (*P* = .796).

The overall rate of not returning to play in GAA athletes who underwent ABR or OL procedures was 12.0% (13.6% vs. 10.3%, respectively; *P* = .52). Of those GAA athletes who did not RTP after ABR and OL procedures, 50% reported that the reason for this was secondary to shoulder-associated factors (7/14 and 5/10, respectively). In addition, the overall rate of RTP while changing sport in GAA athletes who underwent ABR or OL procedures was 2.0% (3.4% vs. 1.0%, respectively; *P* = .621). A summary of these findings is further illustrated in Table III.

**Table III:** Return to play.

Procedure	ABR	OL	*P* value
N Total	103	97	N/a
RTP (%)	89 (86.4)	87 (89.7)	.520
RTP S/H (%)	74 (71.8)	76 (78.4)	.861
RTP time (mo)	5.7	6.2	.240
SIRSI ± SD	86.7 ± 19.4	85.9 ± 24.1	.796
Change sport (%)	3 (3.4)	1 (1.0)	.621
Shoulder issue	3 (100)	0 (0)	.247
Other factor	0 (0)	1 (100)	.485
Did not return (%)	14 (13.6)	10 (10.3)	.520
Shoulder issue	7 (50)	5 (50)	>.99
Other factor	7 (50)	5 (50)	>.99

*ABR*, arthroscopic Bankart repair; *OL*, open Latarjet; *SD*, standard deviation; *RTP*, return to play; *SIRSI*, Shoulder Instability–Return to Sport after Injury.

## Discussion

The most important findings in this study was that GAA athletes with anterior shoulder instability treated with either ABR or OL procedures were found to have excellent clinical outcomes at medium-term follow-up, with excellent functional outcomes and high rates of RTP. In addition, nearly all GAA athletes were satisfied with their outcomes after stabilization with satisfactory subjective shoulder function. Furthermore, this study found that GAA athletes had low recurrence rates after stabilization, with low revision rates in the medium term.

GAA games including Gaelic football and hurling represent the national sports of Ireland. Although GAA games are the most commonly played sports on the island, a longstanding tradition of emigration abroad has led to extensive spread of GAA games to every corner of the globe, with teams established in the United States, United Kingdom, and Australia amongst others, leading to nearly 3000 clubs of GAA globally.^26^ GAA games have previously been described as “high-intensity, high-velocity multidirectional” sports, which subject their athletes to the risks of being a part of both collision and overhead sports, with high rates of injuries occurring.^17^ Previous literature has reported that the incidence of injury for GAA athletes may be as high as 61 injuries per 1000 match hours, with lower limb injuries representing the most common injuries seen in GAA players.^21^^,^^23^^,^^24^ However, similar to other collision sports, shoulder injuries commonly occur during GAA games particularly while tackling or reaching for the ball overhead, as the shoulder is commonly forced into abduction and external rotation, representing a harbinger for potential anterior shoulder instability.^3^ The high incidence of injuries among GAA athletes is not without morbidity. A previous study by O’Connor et al found that of the GAA athletes who suffer injuries, one-third will require radiological assessment, and one-eight will require surgical intervention.^23^

When the athlete elects for surgical intervention of a sport-acquired injury, their primary question often centers on potential RTP.^19^ A previous systematic review by Memon et al reported a pooled rate of RTP of approximately 80% for all athletes after ABR, with nearly two-third returning at preinjury level.^18^ However, in the only previous study reporting outcomes of RTP of GAA athletes after ABR, Clesham and Shannon reported overall RTP rates of approximately 90% after ABR, with over three-quarter returning to play at preinjury level.^5^ These findings are supported by the results of our study, which found high rates of overall RTP and RTP at preinjury levels after ABR. Therefore, the authors believe that ABR represents a viable option for the GAA athlete hoping to RTP in the setting of anterior shoulder instability.

Previous literature has found that the OL procedure results in high rates of RTP for collision and overhead athletes suffering from anterior shoulder instability.^4^^,^^7^ In their systematic review, Hurley et al reported pooled rates of RTP of 88%, with nearly three-quarter returning at preinjury levels after stabilization with the OL procedure.^14^ The results of our study echo these findings, with an RTP rate of nearly 90% for the GAA players included in this study.

The authors of this study believe further study is warranted in relation to time to RTP for the GAA athlete with anterior shoulder instability. Although this study found GAA athletes required approximately 6 months after stabilization to RTP, there is sparse evidence at present reporting such outcomes. In the aforementioned study by Clesham and Shannon, 28 of 31 GAA athletes managed to RTP at approximately 9 months after ABR.^5^ Although the findings of our study add to the encouraging data reported by Clesham and Shannon for GAA athletes after ABR in relation to time to RTP, further study of the area is warranted, as our study remains the only study in the literature reporting such outcomes of GAA athletes treated with OL procedure, with only 6 months required to RTP. Moreover, a recent study by O’Connor et al found that approximately half of all GAA players consider themselves not to be psychologically ready to RTP, despite being cleared clinically.^24^ With all factors considered, the authors believe that perhaps consensus to inform on criteria for RTP in GAA games after surgical stabilization would be of benefit to both orthopedic surgeons in future.

Recurrence rates are of utmost importance for the patient with anterior shoulder instability, with varying figures reported in the literature. In their previous systematic review, Murphy et al reported recurrence in over 30% of patients after ABR.^20^ However, Clesham and Shannon reported a recurrence rate of approximately 15% in GAA athletes after ABR, with no significant difference reported between GAA athletes and athletes of other sports.^5^ However, this study found recurrence rates of approximately 5% for GAA athletes who underwent either ABR or OL procedure for anterior shoulder instability, with the majority of recurrence occurring on the GAA pitch for players who returned to play. However, we note the nonsignificantly higher recurrence rate reported in the GAA athletes who underwent OL vs. ABR procedures, with higher rates of RTP at preinjury level seen in those who underwent OL procedure, as many represent high-performance intercounty athletes whom expressed that RTP represents their most important outcomes preoperatively, in spite of high levels of bone loss. Furthermore, this study found that 98% of all GAA athletes who underwent either ABR or OL procedure were satisfied with their outcomes at over 4 years of mean follow-up, which demonstrates the overall promising clinical findings of this study for GAA athletes in the medium term after shoulder stabilization.

### Limitations

As this study is a retrospective cohort study, it possesses several limitations and sources of potential bias. The primary limitation of the study was that although many clinical outcome measures have been used, included GAA athletes in this study did not have preoperative outcome scores. Furthermore, this study did not use a control group with GAA players with anterior shoulder instability managed conservatively. In addition, although this study compares GAA athletes who underwent ABR and to those who underwent OL procedures in the setting of anterior shoulder instability, this study did not report the percentage of glenoid bone loss calculated preoperatively. Finally, although all GAA games are amateur in nature, they are played at a variety of competitive levels from junior club to intercounty senior level. This has the potential to be a confounding variable, as the level of participation was not taken into account as a patient demographic, but just in relation to RTP analysis.

## Conclusion

GAA athletes with anterior shoulder instability treated with either ABR or OL procedures report excellent clinical outcomes at medium-term follow-up, with high satisfaction rates, excellent functional outcomes, and high rates of RTP. Furthermore, this cohort demonstrates low rates of recurrence after stabilization, with few requiring revision surgery.

## Disclaimers

Funding: No funding was disclosed by the authors.

Conflicts of interest: The authors, their immediate families, and any research foundation with which they are affiliated have not received any financial payments or other benefits from any commercial entity related to the subject of this article.
